# Umbrella Systematic Review of the Efficacy and Safety of PD-1 Inhibitors Combined with CTLA-4 Inhibitors in the Treatment of Melanoma

**DOI:** 10.3390/ijms27093869

**Published:** 2026-04-27

**Authors:** Zhihan Zhou, Chen Zhu, Qifeng Yang, Chenrui Ji, Lihao Ma, Fucai Wang

**Affiliations:** School of Medicine, Huaqiao University, Quanzhou 362021, China; zhzhoumed@163.com (Z.Z.); 2126203046@stu.hqu.edu.cn (C.Z.); 2334132012@stu.hqu.edu.cn (Q.Y.); 2334132005@stu.hqu.edu.cn (C.J.); 2134131015@stu.hqu.edu.cn (L.M.)

**Keywords:** PD-1 inhibitor, CTLA-4 inhibitor, melanoma, umbrella system review, meta-analysis, evidence

## Abstract

The objective is to assess the effectiveness and safety of combining PD-1 inhibitors with CTLA-4 inhibitors for melanoma treatment, drawing on current meta-analysis findings and evaluating the supporting evidence. We used medical subject words and free text words (such as “PD-1 inhibitor”, “CTLA-4 inhibitor”, “melanoma”) as search keywords to search the literature in six literature databases from the establishment of the database to 11 April 2025. Using the PICO (Participant, Intervention, Control, and Outcome) framework, we identified 27 unique associations between combination treatment efficacy outcomes and 70 unique associations between adverse event outcomes, which were re-evaluated using a random effects model. A total of 10 meta-analysis were included, including 36 randomized controlled trials and two retrospective studies. According to the evaluation meta-analysis of AMSTAR 2 (A MeaSurement Tool to Assess Systematic Reviews, version 2), all 10 meta-analysis were of very low quality. The association of outcome indicators was re-analyzed based on the random effects model, of which 26 associations showed high efficacy of combination therapy and 66 associations showed poor safety of combination therapy. In conclusion, a PD-1 inhibitor combined with a CTLA-4 inhibitor is a very effective method for the treatment of melanoma, but the incidence of various types of adverse reactions is high, and the evidence is not reliable. Therefore, future studies need higher quality evidence.

## 1. Introduction

Melanoma, which originates from melanocytes, is a highly aggressive form of skin cancer. Although it accounts for only 1% of all skin cancers, it is responsible for over 75% of skin cancer-related deaths [1], and its incidence is rising globally. While melanoma predominantly affects the elderly, studies have indicated an increasing prevalence among younger populations [2]. Surgical intervention remains the primary treatment for early-stage melanoma, which is often curable through surgery. In contrast, advanced melanoma is mainly treated with immunotherapy, targeted therapy and chemotherapy. For the development of targeted therapies for advanced melanoma, a multicenter randomized controlled trial demonstrated that dabrafenib plus trametinib was active and had controllable safety in patients with BRAF (V600) mutations and brain metastases from melanoma, thereby improving clinical prognosis [3]. In the field of immunotherapy, a multicenter randomized controlled trial indicated that first-line nivolumab combined with ipilimumab or nivolumab alone could provide durable and sustained survival benefits for patients with advanced melanoma, such as the median progression-free survival of the nivolumab plus ipilimumab group was 11.5 months (95% CI 8.7–19.3), whereas that of the nivolumab group was 6.9 months (5.1–10.2), and that of the ipilimumab group was 2.9 months (2.8–3.2) [4].According to the latest data from this study, compared with the sole use of ipilimumab, both nivolumab combined with ipilimumab and nivolumab monotherapy can consistently provide survival benefits to patients with advanced melanoma [5].

With the advancement of research, the emergence of immune checkpoint inhibitors (ICIs), such as cytotoxic T-lymphocyte-associated antigen-4 (CTLA-4), anti-programmed cell death-1 (PD-1), and its primary ligand (PD-L1) has significantly improved treatment prospects for patients with advanced melanoma [4,5,6,7,8,9]. PD-1 is expressed on the surface of antigen-stimulated T cells. Its signal inhibits the activation, proliferation and cytokine secretion of T cells by weakening the downstream pathways of the T cell receptor. Under normal conditions, PD-1 expression rapidly decreases after antigen clearance. However, in environments with continuous antigen exposure, such as tumors, PD-1 remains highly expressed, ultimately leading to the exhaustion of T cells [10]. In recent years, the use of antibodies targeting PD-1/PD-L1 has made significant progress in clinical oncology [5,11].CTLA-4 is expressed by regulatory T cells (Tregs) and acts as a competitive antagonist to CD28-CD80/86 binding, which further impedes the initiation and activation of T cells [12]. CTLA-4 plays a significant role in inhibiting the progression of melanoma. The distinct mechanisms of action of PD-1 and CTLA-4 inhibitors contribute to enhanced anti-tumor effects. PD-1 blockade primarily targets inhibitory signals within the tumor microenvironment, whereas CTLA-4 blockade enhances the immune response at the level of T cell activation. This indicates that the two have complementary effects that optimize T cell activity against tumor cells [13]. Therefore, the combination therapy of different types of ICIs is increasingly becoming a research hotspot in the treatment of melanoma. Existing research has found that in the treatment of melanoma, the use of ICIs often leads to the development of tumor resistance [14,15]. However, numerous RCTs (randomized controlled trials) have demonstrated that ICI combination therapy is superior to single-agent therapy, as it significantly improves patient survival in various cancers, including colorectal cancer [5,16]. However, a meta-analysis by Hao et al. [17] indicated that the combination of nivolumab and ipilimumab did not significantly increase the 1-year overall survival (OS) compared to ipilimumab monotherapy. This might be due to the fact that the number of events in some of the original studies was relatively small at the one-year mark, resulting in a wider confidence interval after the data was combined, and the outcome was not significant. Mearns et al. [18] found that the combination of CTLA-4 and PD-1 inhibitors was more likely to lead to gastrointestinal adverse events and also increased the incidence of potential immune-related adverse events (irAEs) [19]. Moreover, CTLA-4 inhibitors were associated with a higher risk and more severe incidence of immune-related adverse events compared to other ICIs [20]. Additionally, other studies have found that while combination therapy may exert synergistic effects, its toxicity could also increase exponentially [21,22], suggesting that the safety and efficacy of combination therapy require further in-depth research.

Umbrella reviews, often referred to as systematic reviews of systematic reviews, are a research approach that systematically assesses all systematic reviews related to a particular medical research subject to draw more dependable conclusions [23]. This study involved an umbrella review of PD-1 inhibitors combined with CTLA-4 inhibitors in the treatment of melanoma, with the aim of systematically summarizing the research findings and assessing the accuracy of the evidence. This methodology offers an in-depth insight into the effectiveness of combination therapies for melanoma and the relationships among different adverse effects. The main target readers of this umbrella systematic review are researchers, methodologists, and clinical decision support personnel who are engaged in or concerned with systematic reviews and evidence-based medicine for melanoma, including oncologists, clinical epidemiologists, and health technology assessors. For clinicians, the focus of this article lies in presenting the quality and credibility of the existing evidence base, rather than providing specific diagnostic and therapeutic recommendations.

## 2. Materials and Methods

We report the results of this umbrella review in accordance with the PRISMA (Preferred Reporting Items for Systematic Reviews and Meta-Analyses) 2020 statement [24]. The study protocol was registered with the International Prospective Register of Systematic Reviews (PROSPERO, No. CRD420251026820).

### 2.1. Literature Search Strategy

We conducted a comprehensive search across six electronic databases, including PubMed, Embase, Cochrane Library, Web of Science, CNKI, and Wanfang, for literature related to meta-analysis on the combined use of immune checkpoint inhibitors (PD-1 inhibitors, and CTLA-4 inhibitors) in the treatment of melanoma. This search covered the inception of each database up until 11 April 2025, employing the search strategies outlined in Table S1. Two independent researchers initially screened the retrieved literature based on titles and abstracts, followed by a full-text review of the selected studies, with inclusion criteria strictly applied. Any discrepancies in the screening results were resolved through discussion between the two researchers or with the assistance of a third researcher.

### 2.2. Literature Screening and Inclusion Criteria

The inclusion criteria were as follows: (a) meta-analysis published in Chinese or English that examined the combination therapy of immune checkpoint inhibitors for melanoma, with no restrictions on the design or methods of the primary studies included. Furthermore, original studies such as RCTs and in vitro animal experiments were excluded; (b) the intervention involved patients receiving a combination of two different types of immune checkpoint inhibitors, while the control group consisted of patients receiving a single type of immune inhibitor; (c) articles must provide data on outcomes such as OS, objective response rate (ORR), progression-free survival (PFS), and/or related adverse reactions, reporting results in terms of hazard ratio (HR), odds ratio (OR), or relative risk (RR); (d) in cases where multiple meta-analyses involved the same patients, drugs, and outcome measures, one dataset was selected for the pooled effect estimate. Our study excluded articles that lacked full text, available data, or a summarized analysis of relevant associations; (e) this umbrella review only included the meta-analyses evaluating nivolumab + ipilimumab, and did not include data for other combinations (such as pembrolizumab + ipilimumab); and (f) for meta-analyses involving multiple types of solid tumors, only the data from the melanoma subgroup were extracted.

### 2.3. Data Extraction

Two researchers screened the relevant literature independently, utilizing titles, abstracts, and full texts for evaluation. They also extracted data individually using a standardized data collection form, with any discrepancies addressed by consulting a third researcher. For each eligible meta-analysis, the following data were collected: the first author, publication year, type of study population, number of primary studies, primary study design, sample size, intervention, control group, methodological quality assessment tool, treatment effectiveness outcomes, adverse reaction outcomes, effect estimates, and corresponding 95% confidence intervals, along with other analyses. For every primary study that was part of the meta-analysis, we gathered the title, the first author’s name, the year of publication, and the HRs, ORs, or RRs along with their 95% confidence intervals related to the effectiveness of the treatment outcomes. We also recorded the count or occurrence of each adverse event. If the information given in the published articles was inadequate for synthesizing evidence, the corresponding author was contacted via email. Two researchers then reviewed all the data that had been extracted. All adverse events were extracted for all grades, and if different time points of OS were reported in the article, the 1-year or median OS was extracted.

### 2.4. Methodological Quality Assessment

Two researchers independently conducted a methodological quality assessment using AMSTAR 2 (A MeaSurement Tool to Assess Systematic Reviews, version 2) [25], which has demonstrated good consistency, reliability, construct validity, and feasibility. AMSTAR 2 comprises seven critical items and nine non-critical items, categorizing the quality of meta-analyses as high, moderate, low, or critically low. Cohen’s kappa coefficient (κ) was employed to measure inter-rater agreement, and all discrepancies were resolved through discussion with a third researcher.

### 2.5. Data Synthesis and Analysis

Based on the participants, interventions, control groups, and outcomes (PICO) identified in each eligible meta-analysis, a series of unique associations between the efficacy and adverse reactions of PD-1 inhibitors combined with CTLA-4 inhibitors were established. The primary studies included in these meta-analyses were thoroughly reviewed. For each unique association, a meta-analysis was re-conducted using the random effects model. RR, HR, or OR along with their 95% CI were employed to evaluate dichotomous variables, with a significance threshold set at *p* < 0.05. In cases where data from the included primary studies were unavailable, the original effect estimates from the meta-analysis were retained. The heterogeneity of each meta-analysis was assessed using the I^2^ statistic, where an I^2^ value greater than 50% indicates significant heterogeneity. Publication bias was evaluated using Egger’s test, with a *p*-value less than 0.1 indicating significant publication bias. A sensitivity analysis of all associations was conducted using a fixed-effects model. All statistical analyses were performed using R software (version 4.4.1).

### 2.6. Evaluation of the Credibility of Evidence

Apply the GRADE (Grading of Recommendations, Assessment, Development, and Evaluation) criteria to assess the certainty of evidence for each outcome [26]. The certainty of evidence is categorized as high, moderate, low, or very low and may be downgraded due to several factors: risk of bias [27], inconsistency of results [28], indirectness of evidence [29], imprecision [30], and publication bias [31]. The evidence rating is classified as high quality (no downgrades), moderate quality (downgraded by one level), low quality (downgraded by two levels), and very low quality (downgraded by three levels).

### 2.7. Ethical Approval and Patient Consent

The data included in the meta-analyses of this umbrella systematic review were derived from previously published articles and did not require ethical approval. As all articles incorporated into this umbrella systematic review were previously published, the necessity for patient consent was waived.

## 3. Results

### 3.1. Database Search

**Figure 1:**
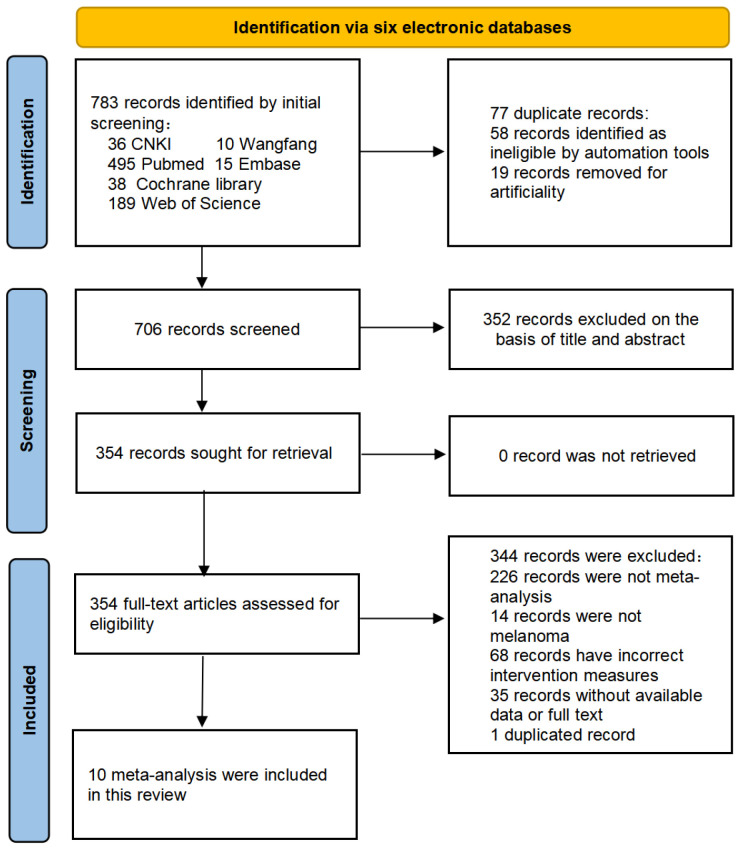
Flow chart of research screening scheme.

A total of 783 articles were retrieved from six literature databases. After removing duplicate records, 706 articles were screened. Based on the titles and abstracts, 352 articles were excluded, leaving 354 articles to be assessed for eligibility against the inclusion criteria. Among these, 344 articles were excluded for various reasons: 226 articles did not conduct a meta-analysis, 14 articles did not focus on melanoma patients, 68 articles did not involve interventions combining immune checkpoint inhibitors, 35 articles lacked available data or full texts, and one article was a duplicate (Figure 1). Ultimately, 10 eligible meta-analysis articles were included, comprising six in English and four in Chinese. The publication years of all included meta-analyses range from 2017 to 2023.

### 3.2. Characteristics of Included Meta-Analysis

The descriptive characteristics of the included meta-analyses are presented in Table 1. Among the 10 meta-analyses, the first authors of eight were from China, one from Egypt, and one from Malaysia. A total of 36 RCTs and two retrospective studies were included across the 10 meta-analyses. The number of RCTs assessing the efficacy and adverse effects of immune checkpoint inhibitor combination therapy in each meta-analysis ranged from 2 to 9. Furthermore, among the eligible meta-analyses, one included two retrospective studies [32], while none included prospective studies. The sample sizes of the 10 eligible meta-analysis varied from 1087 to 4150. The efficacy outcome measures of the studies included: ORR, PFS, OS, DCR, and PRR. The common and important adverse events of immune checkpoint inhibitors are well-known in oncology clinical practice [33,34]. Adverse health consequences encompass a variety of mixed adverse reaction outcomes and involve reactions affecting different organ systems. See Table 1 for details. The subjects of this study included those with unresectable stage III/IV melanoma or brain metastasis. Early clinical trials usually excluded patients with brain metastases, but a study [35] has shown that immune checkpoint inhibitors are effective in some patients with brain metastases. Therefore, this umbrella review includes studies involving such patients to be more in line with current clinical practice. In the ten meta-analyses reviewed, the experimental group received a combined treatment of nivolumab (PD-1 inhibitor) and ipilimumab (CTLA-4 inhibitor), while the control group was treated with nivolumab and/or ipilimumab or their placebo.

Among the 10 meta-analyses, all 10 reported the methodological quality assessment of the primary studies, with nine utilizing the Cochrane Risk of Bias tool and 1 employing the Jadad scale.

In the 10 meta-analyses, 27 associations concerning the effectiveness outcomes of combination therapy and 70 associations related to adverse reaction outcomes were described and analyzed. Notably, the adverse reaction data from Li et al. [36], Hao et al. [17] and Zeng et al. [41] could not be re-analyzed; therefore, the original data were utilized directly. Among the 27 associations pertaining to effectiveness outcomes, sample sizes varied from 19 to 2535, with eight associations demonstrating substantial heterogeneity (I^2^ > 50%). For the 70 associations related to adverse reaction outcomes, sample sizes ranged from 202 to 3421, with 13 associations exhibiting substantial heterogeneity (I^2^ > 50%).

### 3.3. Methodological Quality of Included Meta-Analysis

Based on the findings from the methodological quality assessment utilizing AMSTAR 2, all 10 meta-analyses (100%) exhibited critically low methodological quality, with none classified as having high, moderate, or low confidence. The two researchers involved in the study reached a high level of consensus on the evaluation outcomes (κ = 0.69); comprehensive details can be found in Table 2.

### 3.4. Effectiveness Outcomes

**Figure 2:**
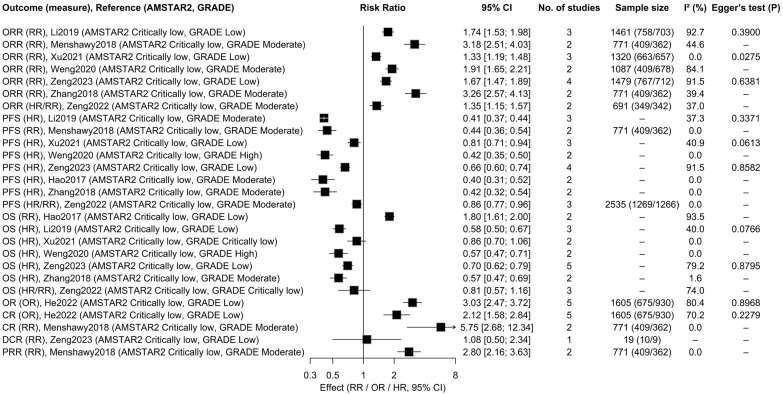
Efficacy outcomes of combined treatment with immune checkpoint inhibitors in patients. References included in this figure: [17,32,36,37,39,40,41,42,43].

According to the random effects model, 24 out of 27 associations (88.9%) were statistically significant. Among the 27 associations, two (8.3%) exhibited high credibility of evidence, 12 (50.0%) demonstrated moderate credibility of evidence, 11 (45.8%) indicated low credibility of evidence, and two (8.3%) had very low credibility of evidence (Figure 2 and Table S2). Among the 24 statistically significant associations, regarding the association with ORR, seven studies [36,37,39,40,41,42,43] consistently demonstrated that the combination therapy of nivolumab plus ipilimumab was superior to monotherapy, significantly improving the ORR in melanoma patients. Notably, four of these studies [37,39,41,43] were supported by moderate certainty evidence: among them, Menshawy et al. [37] found that the combination therapy further enhanced ORR for patients with advanced stage III or IV melanoma (RR, 3.18; 95% CI, 2.51–4.03). Regarding the association with PFS, eight studies [17,36,37,39,40,41,42,43] indicated that the combination therapy significantly improved patients’ PFS and reduced the risk of disease progression or death. Of these, one study [39] was supported by high certainty evidence, demonstrating that for melanoma patients, the risk of disease progression or death in the combination therapy group was 58% lower than in the monotherapy group (HR, 0.42; 95% CI, 0.35–0.50). Five studies [36,39,40,42,43] demonstrated that combination therapy significantly improved OS in patients. One study [39], supported by high-quality evidence, indicated that for melanoma patients, the combination therapy group had a 43% lower risk of disease progression or death compared to the monotherapy group (HR, 0.57; 95% CI, 0.47–0.71). Another study was supported by moderate-quality evidence, while the remaining study reported an OS risk ratio of 1.80 (95% CI, 1.61–2.00) [17]. However, there was significant heterogeneity among the studies (I^2^ = 93.50%). Additional details are presented in Figure 2.

Among the three associations that were not re-analyzed due to lack of statistical significance or absence of primary study raw data, and were directly extracted from the original text, two studies indicated that combination therapy improved patients’ OS [40,41], while one study suggested that combination therapy enhanced patients’ DCR [42].

### 3.5. Adverse Outcomes

**Figure 3:**
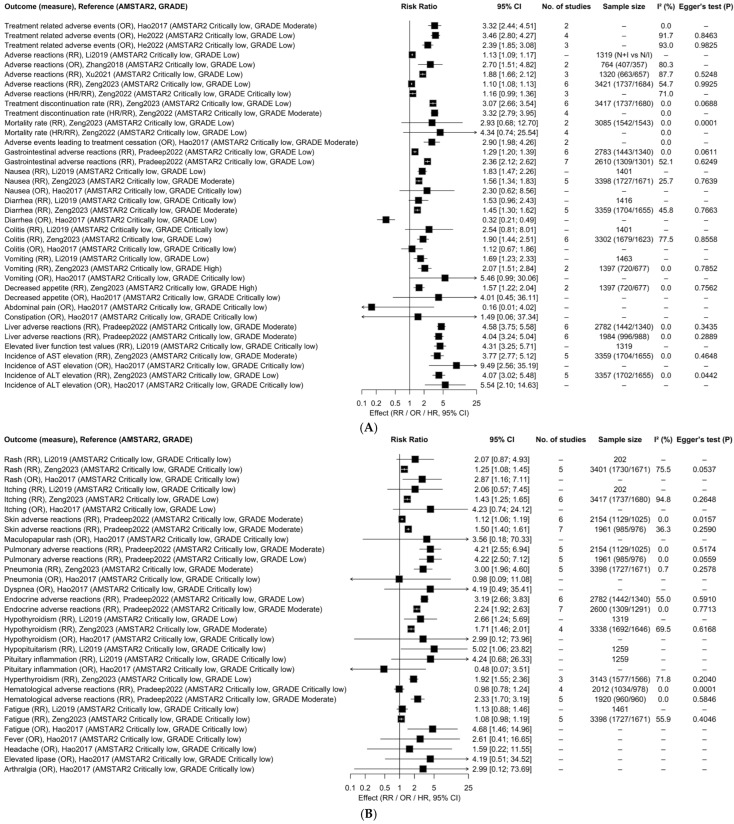
(**A**,**B**) Adverse reaction outcomes of combined treatment with immune checkpoint inhibitors in patients. References included in this figure: [17,32,36,38,40,41,42,43].

According to the random effects model, 38 out of 70 associations (54.3%) were found to be statistically significant. Among the 70 associations, two (2.9%) had high credibility of evidence, 15 (21.4%) had moderate credibility of evidence, 22 (31.4%) had low credibility of evidence, and 31 (44.3%) had very low credibility of evidence (Figure 3 and Table S3). Among the 38 statistically significant associations, all studies indicated that combination therapy increased the risk of adverse outcome events. Regarding gastrointestinal adverse outcomes, two studies [38] indicated an increased risk of gastrointestinal adverse reactions, with moderate certainty of evidence supported by two studies [38]. Additionally, two studies [36,42] demonstrated an increased risk of nausea, with moderate certainty of evidence supported by one study [42]. Furthermore, two studies [36,42] illustrated an increased risk of vomiting, with high certainty of evidence supported by one study [42]. For patients receiving combination therapy, the risk of vomiting was found to be higher (RR, 2.07; 95% CI, 1.51–2.84). However, the absolute incidence rate is very low, so in clinical management, attention should be paid to this aspect, but it is not the main toxic burden. Other descriptions are presented in Figure 3.

### 3.6. Sensitivity Analysis

Taking into account the varying demographic characteristics among the studies included, a fixed effects model was utilized for a sensitivity analysis to assess the reliability of the aggregated findings. In the efficacy outcomes of patients receiving combined immune checkpoints inhibitors therapy, the majority of associations (25 associations; 92.6%) were consistent with the main analysis results, while the remaining two studies showed statistical significance when a random effects model was applied but not when a fixed-effects model was used. In the adverse reaction outcomes of patients receiving combined immune checkpoints inhibitors therapy, the majority of associations (67 associations; 95.7%) were consistent with the main analysis results, while the remaining three studies showed statistical significance when a random-effects model was applied but not when a fixed-effects model was used. Overall, according to the fixed-effects model, the efficacy of combination therapy is favorable but still associated with significant safety concerns. Specific results are presented in Tables S2 and S3.

## 4. Discussion

Immune checkpoint inhibitors, particularly the combination of anti-PD-1 and anti-CTLA-4 therapies, have been established as a standard of care for advanced melanoma over the past decade, with their efficacy and safety profiles extensively characterized in randomized controlled trials and high-quality research [5,44,45]. Therefore, a comprehensive synthesis and critical appraisal of the existing evidence is essential. In this context, our umbrella review systematically evaluates the efficacy and safety of combination immunotherapy by assessing the methodological quality and credibility of existing meta-analyses using the AMSTAR 2 and GRADE frameworks. A total of 10 meta-analyses were included, comprising 36 randomized controlled trials and two retrospective studies. Importantly, although a substantial body of clinical evidence is available, our findings indicate that the methodological quality of existing meta-analyses is critically low, which limits the reliability of their conclusions. Therefore, rather than providing a definitive summary of efficacy and safety, this study offers a structured and critical synthesis of the current evidence base, with the aim of informing future high-quality research and improving evidence synthesis in this field. The relevant conclusions should be interpreted in conjunction with original high-quality RCTs and the latest long-term data.

The quality of the meta-analyses was evaluated using AMSTAR 2, and the results indicated an overall low credibility, which severely limits the ability to draw definitive conclusions regarding the efficacy and safety of PD-1 inhibitors combined with CTLA-4 inhibitors in the treatment of melanoma patients. This is primarily due to the following reasons: (1) many articles lack protocol registration or registration information, which is crucial for reducing the risk of bias in the systematic review process and enhancing the rigor and transparency of systematic reviews; (2) none of the included articles analyzed the reasons for including RCTs; (3) few articles provided a list of the excluded literature and explained the reasons for exclusion, thereby reducing the transparency and objectivity of the literature screening; (4) although commercially funded research projects are more likely to yield positive results for the funded products, none of the included articles summarized the funding sources of the studies included in the analysis; (5) the included meta-analyses did not adequately discuss studies with moderate or high risk of bias, nor did they explain the potential impact of such biases on the study results; (6) many meta-analyses failed to adequately explain or explore the sources of heterogeneity in the study results, thereby diminishing the credibility of their findings; and (7) the assessment of publication bias was not conducted, and numerous articles failed to incorporate grey literature, unpublished research, and ongoing trials, potentially impacting the accuracy of the conclusions drawn. These identified gaps should be rectified in forthcoming meta-analyses.

In this umbrella review, we identified 27 associations concerning efficacy outcomes. Among these, most studies suggested that combination therapy may improve efficacy indicators such as ORR, PFS, OS, DCR, and PRR. Based on these findings, the combination of anti-PD-1 and anti-CTLA-4 immunotherapy may represent an effective treatment approach for advanced melanoma, although the certainty of evidence remains limited. Regarding the safety profile of combination therapy, previous studies have shown that the toxicities associated with the use of single-agent ICIs manifest as myocarditis, shortness of breath, palpitations, edema, and fatigue [46]. Patients receiving dual ICI treatment may experience a significantly higher incidence of irAEs compared to those on monotherapy [47]. Our study identified 70 unique associations, among which 66 (94.3%) studies consistently demonstrated that combination therapy increases the risk of adverse reactions across various organs. The spectrum of these adverse events ranges from dermatitis and colitis to more severe complications such as pneumonitis and endocrine disorders, all of which necessitate careful monitoring and management strategies. Regarding gastrointestinal adverse reactions, two studies indicated an increased risk of such events, both supported by evidence of moderate certainty; two studies indicated an increased risk of nausea, one of which was supported by evidence of moderate certainty; and two studies indicated an increased risk of vomiting, one of which was supported by evidence of high certainty. In terms of hepatic adverse reactions, both studies showed that the incidence of elevated AST and ALT was significantly higher in combination therapy compared to monotherapy. A study by Zeng et al. [42] demonstrated a significantly increased incidence of elevated AST (RR, 3.77; 95% CI: 2.77–5.12). Regarding skin adverse reactions, multiple studies have shown that combination therapy significantly increases the likelihood of skin adverse reactions, such as rash, pruritus, and maculopapular rash. In terms of pulmonary adverse reactions, four studies have indicated that the likelihood of adverse reactions, including pneumonitis and dyspnea, is greater in the combination therapy group compared to the monotherapy group. Therefore, when considering the anti-PD-1 combined with anti-CTLA-4 treatment regimen for melanoma, it is essential to consider additional drug combinations to mitigate these adverse events. Future research should explore strategies to mitigate irAEs without compromising efficacy, such as using anti-inflammatory modulating agents [48]. Moreover, oncologists should focus on common and clinically significant immune-related adverse events, such as colitis, hepatitis, rash, endocrine abnormalities, etc. These occur frequently in combination therapy and have a significant impact on the continuity of treatment. Rare events (such as vomiting, severe hematological toxicity) are statistically significant, but due to their low absolute risk, they have limited weight in clinical decision-making. However, some associations were based on only one RCT, and the limited evidence may lead to systematic bias. Other non-significant associations face similar challenges. Only two associations (2.9%) were supported by high-confidence evidence, while 15 associations (21.4%) had moderate-confidence evidence. Overall, there is a lack of high-quality evidence supporting the safety of combination therapy regimens. Additionally, it is suggested that the occurrence of early and late adverse reactions in patients undergoing immunotherapy and immunoradiotherapy for solid tumors can be viewed as favorable prognostic parameters, especially when these events are delayed, as they are associated with increased overall response rates and improvements in OS and PFS in these patients [49,50]. These findings highlight the importance of careful monitoring and management of immune-related adverse events in clinical practice.

This umbrella review presents several advantages. First, systematic searches were carried out independently by two researchers across six electronic databases to collect up-to-date evidence regarding the effectiveness of combination therapy using PD-1 and CTLA-4 inhibitors, along with its correlations to different adverse health outcomes, which were examined based on reactions in various organs, thus improving the article’s organization and clarity. Second, we strictly adhered to the back-to-back principle for the literature selection, data extraction, and evaluation of methods and evidence quality. Third, the majority of data included in this umbrella review stemmed from RCTs, thereby enhancing the quality of the original evidence based on the findings from the literature search and selection. Finally, where applicable, we adopted a standardized methodology utilizing the random-effects model for performing meta-analyses on each identified association. The I^2^ statistic was recalculated to evaluate heterogeneity, and Egger’s test was conducted to determine the likelihood of publication bias. This method allows for improved comparison of various outcomes and strengthens the assessment of the evidence’s reliability.

However, this study has several limitations. First, the literature search was confined to English and Chinese databases, which may have introduced language bias. Second, this umbrella review was based solely on existing meta-analyses and did not evaluate any new trials published thereafter that assessed the efficacy and safety of combination therapies with immune checkpoint inhibitors, potentially leading to data omission and affecting the results. Third, the majority of the included meta-analysis exhibited low evidence and methodological quality, which may reduce the credibility of the article’s conclusions. Therefore, they cannot be used as the sole basis for clinical decision-making. However, they remain the main available forms of evidence integration at present, and their quality issues precisely highlight the necessity of conducting high-quality systematic reviews and original research in the future. Fourth, although different doses of immune checkpoint inhibitors could influence outcomes, a dose–response meta-analysis was not conducted in this review due to the lack of necessary information in most meta-analysis. Fifth, this review did not conduct a repeated quality assessment of the primary studies, which poses a risk of reporting bias in the meta-analysis. Instead, the quality of the primary studies was based on the evaluation results reported by the original authors. The inclusion of a small number of trials in some subgroups makes the results more susceptible to potential publication bias. Due to the limited number of studies in each subgroup analysis, we could not confidently assess publication bias. Sixth, although several RCTs are currently reporting on other combination regimens [51,52], this article does not include studies on other ICI combination treatments, such as the meta-analysis of the combination therapy of nivolumab and relatlimab. The number of such meta-analyses is too small, indicating that in the future, when relevant systematic reviews and meta-analyses are available, they can be included in the umbrella analysis to update the evidence and conduct more comprehensive and systematic research to further understand the prognosis of different combination regimens.

## 5. Conclusions

This umbrella review systematically evaluated the efficacy and safety of combined PD-1 and CTLA-4 inhibitor therapy in melanoma by critically appraising existing meta-analyses. The findings suggest that nivolumab combined with ipilimumab therapy may be associated with improved efficacy outcomes. It has a better therapeutic effect than single treatment and either nivolumab or ipilimumab. Eight meta-analyses demonstrated that the combined treatment significantly prolonged PFS, and five meta-analyses demonstrated that the combined treatment significantly improved OS; however, it is also linked to an increased risk of immune-related adverse events. Importantly, the overall methodological quality and certainty of evidence in the included meta-analyses were critically low, which limits the reliability of these findings. Therefore, current evidence should be interpreted with caution rather than as definitive conclusions. Future high-quality studies and rigorously conducted meta-analyses are needed to provide more reliable evidence regarding the balance between efficacy and safety in combination immunotherapy for melanoma.

## Figures and Tables

**Table 1:** Features of the included meta-analysis.

Reference	Country	Validity Index	Adverse Reaction Index	Intervention	Control	Population	Number of Patients	Studies Included	Methodology Quality Assessment Tool	Prospective Study Included	RCTs Included	Retrospective Study Included
Hao 2017 [17]	China	ORR; PFS; OS	①②③④⑥⑧	N + I	I	Advanced cutaneous melanoma	2174	4	the Cochrane Risk of Bias tool	0	2	0
He 2022 [32]	China	ORR, CR	①	N + I	N/I	Advanced stage III or stage IV melanoma	1605	5	the Cochrane Risk of Bias tool	0	3	2
Li 2019 [36]	China	ORR; PFS; OS	①②③④⑥⑧	N + I	N/I	Advanced or metastatic melanoma	2234	5	Jaded	0	3	0
Menshawy 2018 [37]	Egypt	ORR, CR, PRR, PFS	No	N + I	I	Advanced stage III or stage IV melanoma	1087	2	the Cochrane Risk of Bias tool	0	2	0
Pradeep 2022 [38]	Malaysia	ORR; PFS; OS	②③④⑤⑥⑦	N + I	N/I	Advanced stage III or stage IV melanoma	4150	9	the Cochrane Risk of Bias tool	0	9	0
Weng 2020 [39]	China	ORR, PFS, OS	No	N + I	N/I	Melanoma	1087	2	the Cochrane Risk of Bias tool	0	2	0
Xu 2021 [40]	China	ORR, PFS, OS	①	N + I	N	Advanced melanoma, Melanoma brain metastasis, Untread melanoma	1320	3	the Cochrane Risk of Bias tool	0	3	0
Zeng 2022 [41]	China	ORR, PFS, OS	①	N + I	N	Melanoma, Melanoma brain metastasis	2650	4	the Cochrane Risk of Bias tool	0	4	0
Zeng 2023 [42]	China	ORR, PFS, OS; DCR	①②③④⑤⑥⑧	N + I	N/I/N + I placebo	Melanoma (include advanced melanoma and melanoma brain metastasis)	3125	6	the Cochrane Risk of Bias tool	0	6	0
Zhang 2018 [43]	China	ORR, PFS, OS	①②③④⑥⑧	N + I	I	Stage III or stage IV melanoma	1087	2	the Cochrane Risk of Bias tool	0	2	0

① mixed adverse reaction outcomes (treatment-related adverse events; adverse reaction; treatment discontinuation rate; mortality rate; adverse events leading to treatment cessation); ② gastrointestinal adverse reaction outcomes (gastrointestinal adverse reactions; nausea; diarrhea; colitis; vomiting; loss of appetite; decreased appetite; constipation; ③ liver adverse reaction outcomes (adverse liver reactions; elevated liver function test values; incidence of AST elevation; incidence of ALT elevation; ④ skin adverse reaction outcomes (rash; itching; skin adverse reactions; maculopapular rash); ⑤ pulmonary adverse reaction outcomes (pulmonary adverse reactions; pneumonitis; dyspnea; ⑥ endocrine adverse reaction outcomes (endocrine adverse reactions; hypothyroidism; hypopituitarism; pituitary inflammation; hyperthyroidism); ⑦ hematological adverse reaction outcomes (hematological adverse reactions); ⑧ others (fatigue; fever; headache; elevated lipase; arthralgia. N: Nivolumab; I: Ipilimumab.

**Table 2:** Methodological quality assessment using AMSTAR 2.

Reference	Item																Methodological Quality
	1	2 *	3	4 *	5	6	7 *	8	9 *	10	11 *	12	13 *	14	15 *	16	
Hao 2017 [17]	Y	N	N	PY	Y	Y	N	PY	Y	N	Y	N	N	N	N	N	Critically low
He 2022 [32]	Y	N	N	PY	Y	Y	N	PY	Y	N	Y	N	N	N	N	N	Critically low
Li 2019 [36]	Y	N	N	PY	Y	Y	N	PY	Y	N	Y	N	N	Y	N	Y	Critically low
Menshawy 2018 [37]	Y	N	N	PY	Y	N	N	PY	Y	N	N	N	N	N	N	Y	Critically low
Pradeep 2022 [38]	Y	N	N	PY	Y	Y	Y	PY	Y	N	Y	N	N	N	N	Y	Critically low
Weng 2020 [39]	Y	N	N	PY	Y	Y	N	PY	Y	N	Y	N	N	Y	Y	N	Critically low
Xu 2021 [40]	N	N	N	PY	N	Y	N	PY	Y	N	Y	N	N	N	Y	Y	Critically low
Zeng 2022 [41]	Y	N	N	PY	Y	N	N	PY	Y	N	Y	N	N	N	Y	N	Critically low
Zeng 2023 [42]	Y	N	N	PY	Y	Y	N	PY	Y	N	Y	N	N	Y	Y	N	Critically low
Zhang 2018 [43]	Y	N	N	PY	Y	Y	N	PY	Y	N	Y	N	N	N	Y	N	Critically low

* Refers to acritical items. Y = yes; N = no; PY = partial yes.

## Data Availability

Data: extracted from included studies. No new data were created or analyzed in this study. Data sharing is not applicable to this article.

## Supplementary Materials

The following supporting information can be downloaded at: https://www.mdpi.com/article/10.3390/ijms27093869/s1.

## Abbreviations

The following abbreviations are used in this manuscript:

RCT	randomized controlled trial
OS	overall survival
ORR	objective response rate
PFS	progression-free survival
DCR	disease control rate
PRR	partial response rate
HR	hazard ratio
OR	odds ratio
RR	relative risk
AST	aspartate transaminase
ALT	alanine aminotransferase
AMSTAR 2	A MeaSurement Tool to Assess Systematic Reviews version 2
irAEs	immune-related adverse events
GRADE	Grading of Recommendations, Assessment, Development, and Evaluation
CTLA-4	cytotoxic T-lymphocyte-associated antigen-4
PD-1	programmed cell death-1
PD-L1	programmed cell death ligand-1
ICI	immune checkpoint inhibitors
