# Supplementary material for: Umbrella Systematic Review of the Efficacy and Safety of PD-1 Inhibitors Combined with CTLA-4 Inhibitors in the Treatment of Melanoma
Source: Int J Mol Sci. 2026 Apr 27;27(9):3869. doi: 10.3390/ijms27093869 (PMC13164474; doi:10.3390/ijms27093869)
Supplement: Supplementary file 1 [file ijms-27-03869-s001.zip › ijms-4200862-supplementary.pdf]

Supplementary Material

Database	Search term	Results
PubMed	((((((Meta-Analysis[Publication Type]) OR (Systematic Review[Publication Type])) OR (Meta Analysis)) OR ((Systematic Review)) OR (pooled analysis)) OR (synthesis)) AND ((((((("Immune Checkpoint Inhibitors"[Mesh]) OR (Checkpoint Blockers, Immune[Title/Abstract])) OR (CTLA 4 Inhibitors[Title/Abstract])) OR (Cytotoxic T Lymphocyte Associated Protein 4 Inhibitor[Title/Abstract])) OR (PD 1 Inhibitors[Title/Abstract])) OR (PD-L1 Inhibitors[Title/Abstract]))) AND (((("Melanoma"[Mesh]) OR (Melanomas[Title/Abstract])) OR (Malignant Melanoma[Title/Abstract])) OR (Melanomas, Malignant[Title/Abstract]))	495
Embase	#9 #4 AND #6 AND #7 AND #8<br>#8 #4 AND #6 AND #7<br>#7 melanomas:ab,ti OR 'malignant melanoma':ab,ti OR 'malignant melanomas'ab,ti OR 'melanoma,malignant:ab,ti OR 'melanomas,malignant':ab,ti<br>#6 'meta-analysis[publication type]' it OR 'systematic review(publication type]':it OR 'meta analysis':ab,it OR 'systematic review': ab,ti OR'pooled analysis' ab,ti OR synthesis:ab,ti<br>#5 'meta-analysis[publication type]':it OR 'systematic review[publication type]':it OR 'meta analysis':ab,ti OR 'systematic review':ab,ti OR 'pooled analysis':ab,ti OR synthesis:ab,ti OR melanomas:ab,ti OR 'malignant melanoma':ab,ti OR 'malignant melanomas':ab,ti OR 'melanoma,malignant':ab,ti OR 'melanomas, malignant':ab,ti<br>#4 'immune checkpoint inhibitor':ab,ti OR 'checkpoint inhibitors, immune':ab,ti OR 'ctla-4 inhibitors':ab,ti OR 'cytotoxic t-lymphocyte-associated protein 4 inhibitors':ab,ti OR 'ctla-4 inhibitor':ab,ti OR 'pd-1 inhibitors':ab,ti OR 'programmed cell death protein 1 inhibitor':ab,ti OR 'pd-1'<br>inhibitors':ab,ti OR 'programmed death-ligand 1 inhibitors':ab,ti<br>#3 'immune checkpoint inhibitor':ab,ti OR 'checkpoint inhibitors, immune':ab,ti OR 'checkpoint blockers, immune':ab,ti<br>#2 'pd1'/exp OR pd1<br>#1 'pd1'/exp OR pd1	15
Cochrane Library	#1 MeSH descriptor:[Immune Checkpoint Inhibitors], explode all trees<br>#2 MeSH descriptor:[Melanoma] explode all trees<br>#3 (Meta-Analysis):pt OR (Systematic Review):pt OR (Meta Analysis):ti,ab,kw OR (Systematic Review):ti,ab,kw OR (pooled analysis or synthesis):ti,ab<br>#4 (Melanomas):ti,ab,kw OR (Malignant Melanoma or Malignant Melanomas or Melanoma,Malignant or Melanomas,Malignant):ti,ab,kw<br>#5 (Immune checkpoint inhibitor):ti,ab,kw OR (Checkpoint Inhibitors, Immune or Checkpoint Blockers, Immune or CTLA-4 Inhibitors or Cytotoxic T-Lymphocyte-Associated Protein 4 lnhibitors or PD-1 Inhibitors or Programmed Cell Death Protein 1 Inhibitor or PD-L1 Inhibitors or Programmed Death-ligand 1 Inhibitors)<br>#6 #1 or #5<br>#7 #2 or #4<br>#8 #3 and #6 and #7	38
Web of science	1: (((((TS=(Meta-Analysis)) OR TS=(Systematic Review)) OR TI=(Meta Analysis)) OR TI=(Systematic Review)) OR TI=(pooled analysis)) OR TI=(synthesis)<br>2： (((((TS=(Immune Checkpoint Inhibitors)) OR TS=(checkpoint blockers immune)) OR TS=(ctla 4 inhibitors)) OR TS=(cytotoxic t lymphocyte associated protein 4 inhibitor)) OR TS=(pd 1 inhibitors)) OR TS=(pd l1 inhibitors)<br>3:((((TI=(Melanomas)) OR TI=(Malignant Melanoma)) OR TI=(Malignant Melanomas)) OR TI=(Melanoma, Malignant)) OR TI=(Melanomas, Malignant)<br>4:#1 AND #2 AND #3	189
CNKI	Abstract: PD1+PD1/PD-L1+PD1/PDL1+CTLA-4 + Immune Checkpoint Inhibitors AND Abstract: Meta-analysis + Systematic Review AND Abstract: Melanoma + Malignant Melanoma	36
Wangfang	Title or Keywords: (meta-analysis OR systematic review) AND Title or Keywords: (melanoma OR malignant melanoma) AND Title or Keywords: (immune checkpoint inhibitors OR PD-1 OR PD-L1 OR CTLA-4)	10

Supplementary Table S1. Search strategies

Reference	Population	Intervention	Control	No. of studies	Sample size(Intervention /control)	Metric	Random Effect Size (95% CI)	*P-value*	I^2, %	Egger's Test *(P-value)*	Risk of Bias	Inconsistency	Indirectness	Imprecision	Publication Bias	GRADE Rating	AMSTAR 2 Rating
**ORR**																	
Li2019#(1)	Advanced or metastatic melanoma	N+I	I/N	3	1461 (758/703)	RR	FE:1.74 [1.53;1.98] RE:2.51 [1.38;4.57]	FE:<0.0001 RE:0.0025	FE:92.7% RE:92.7%	0.39	-1	-1	0	0	No	Low	Critically low
Menshawy 2018(2)	Advanced stage III or stage IV melanoma	N+I	I	2	771 (409/362)	RR	FE:3.18 [2.51;4.03] RE:2.51 [1.38;4.57]	FE:<0.0001 RE:0.0025	FE:44.6% RE:92.7%	NA	-1	0	0	0	NA	Moderate	Critically low
Xu2021(3)	Advanced melanoma, Melanoma brain metastasis, Untreated melanoma	N+I	N	3	1320 (663/657)	RR	FE:1.33 [1.19;1.48] RE:1.33 [1.19; 1.48]	FE:<0.0001 RE:<0.0001	FE:0.0% RE:0.0%	0.0275	-1	0	0	0	Yes	Low	Critically low
Weng2020 (4)	Melanoma	N+I	N/I	2	1087 (409/678)	RR	FE:1.91 [1.65;2.21] RE:2.95 [1.02;8.54]	FE:< 0.0001 RE:0.0461	FE:84.1% RE:68.3%	NA	0	-1	0	0	NA	Moderate	Critically low
Zeng2023(5)	Melanoma (include advanced melanoma and melanoma brain metastasis)	N+I	N/I/N+I Placebo	4	1479 (767/712)	RR	FE:1.67 [1.47; 1.89] RE:1.97 [0.93;4.14]	FE:< 0.0001 RE: 0.0749	FE:91.5% RE:91.5%	0.6381	-1	-1	0	0	No	Low	Critically low
Zhang 2018(6)	Stage III or stage IV melanoma	N+I	I	2	771 (409/362)	RR	FE:3.26 [2.57; 4.13] RE:3.58 [2.20;5.83]	FE: <0.0001 RE:<0.0001	FE:39.4% RE:39.4%	NA	-1	0	0	0	NA	Moderate	Critically low
Zeng 2022#(7)	Melanoma, Melanoma brain metastasis	N+I	N	2	691 (349/342)	RR/ HR	FE:1.35 [1.15; 1.57] RE:/	FE: <0.01 RE:/	FE:37% RE:/	/	-1	0	0	0	NA	Moderate	Critically low
**PFS**																	
Li2019#(1)	Advanced or metastatic melanoma	N+I	I/N	3	/	HR	FE:0.41 [0.37; 0.44] RE:0.40 [0.35; 0.45]	FE:< 0.0001 RE:< 0.0001	FE:37.3% RE:37.3%	0.3371	-1	0	0	0	No	Moderate	Critically low
Menshawy 2018(2)	Advanced stage III or stage IV melanoma	N+I	I	2	771 (409/362)	RR	FE:0.44 [0.36; 0.54] RE:0.44 [0.36; 0.54]	FE:< 0.0001 RE:< 0.0001	FE:0.0% RE:0.0%	NA	-1	0	0	0	NA	Moderate	Critically low

Xu2021(3)	Advanced melanoma, Melanoma brain metastasis, Untreated melanoma	N+I	N	3	/	HR	FE:0.81 [0.71; 0.94] RE:0.81 [0.71; 0.94]	FE:0.0050 RE:0.0050	FE:40.9% RE:40.9%	0.0613	-1	0	0	0	Yes	Low	Critically low
Weng2020 (4)	Melanoma	N+I	N/I	2	/	HR	FE:0.42 [0.35;0.50] RE: 0.42 [0.35;0.50]	FE:<0.0001 RE:<0.0001	FE:0.0% RE:0.0%	NA	0	0	0	0	NA	High	Critically low
Zeng2023(5)	Melanoma (include advanced melanoma and melanoma brain metastasis)	N+I	N/I/N+I Placebo	4	/	HR	FE:0.66 [0.60; 0.74] RE:0.64 [0.42; 0.96]	FE:< 0.0001 RE: 0.0321	FE:91.5% RE:91.5%	0.8582	-1	-1	0	0	No	Low	Critically low
Hao2017# (8)	Advanced Cutaneous Melanoma	N+I	N	2	/	HR	FE:0.40 [0.31; 0.52] RE:0.40 [0.31; 0.52]	FE:<0.0001 RE:< 0.0001	FE:0.0% RE:0.0%	NA	-1	0	0	0	NA	Moderate	Critically low
Zhang 2018(6)	Stage III or stage IV melanoma	N+I	I	2	/	HR	FE:0.42 [0.32; 0.54] RE:0.42 [0.32; 0.54]	FE:< 0.0001 RE:< 0.0001	FE:0.0% RE:0.0%	NA	-1	0	0	0	NA	Moderate	Critically low
Zeng 2022#(7)	Melanoma, Melanoma brain metastasis	N+I	N	3	2535 (1269/1266)	HR/RR	FE:0.86 [0.77; 0.96] RE:/	FE:0.007 RE:/	FE:0% RE:/	/	-1	0	0	0	NA	Moderate	Critically low
**OS**																	
Hao2017# (8)	Advanced Cutaneous Melanoma	N+I	N	2	/	RR	FE:1.80 [1.61;2.00] RE:1.53 [0.89;2.64]	FE:<0.0001 RE:0.1220	FE:93.5% RE:93.5%	NA	-1	-1	0	0	NA	Low	Critically low
Li2019#(1)	Advanced or metastatic melanoma	N+I	I/N	3	/	HR	FE:0.58 [0.50;0.67] RE:0.64 [0.48;0.84]	FE:< 0.0001 RE:0.0017	FE:40.0% RE:40.0%	0.0766	-1	0	0	0	Yes	Low	Critically low
Xu2021(3)	Advanced melanoma, Melanoma brain metastasis, Untreated melanoma	N+I	N	2	/	HR	FE:0.86 [0.70; 1.06] RE:0.86 [0.70;1.06]	FE:0.1620 RE:0.1620	FE:0.0% RE:0.0%	NA	-1	0	0	-1	NA	Very Low	Critically low
Weng2020 (4)	Melanoma	N+I	N/I	2	/	HR	FE:0.57 [0.47;0.71] RE: 0.57 [0.47;0.71]	FE:<0.0001 RE:<0.0001	FE:0.0% RE:0.0%;	NA	0	0	0	0	NA	High	Critically low

Zeng2023(5)	Melanoma (include advanced melanoma and melanoma brain metastasis)	N+I	N/I/N+I Placebo	5	/	HR	FE:0.70 [0.62;0.79] RE:0.69 [0.52;0.92]	FE:<0.0001 RE:0.0110	FE:79.2% RE:79.2%	0.8795	-1	-1	0	0	No	Low	Critically low
Zhang 2018(6)	Stage III or stage IV melanoma	N+I	I	2	/	HR	FE:0.57 [0.47;0.69] RE:0.57 [0.47;0.69]	FE:<0.0001 RE:<0.0001	FE:1.6% RE:1.6%	NA	-1	0	0	0	NA	Moderate	Critically low
Zeng 2022#(7)	Melanoma, Melanoma brain metastasis	N+I	N	3	/	HR/ RR	FE:/ RE:0.81 [0.57; 1.16]	FE: / RE:0.25	FE:/ RE:74%	/	-1	-1	0	-1	NA	Very Low	Critically low
**OR***																	
He2022(9)	Advanced stage III or stage IV melanoma	N+I	N/I	5	1605 (675/930)	OR	FE:3.03 [2.47;3.72] RE:3.16 [1.82;5.50]	FE:<0.0001 RE:<0.0001	FE:80.4% RE:80.4%	0.8968	-1	-1	0	0	No	Low	Critically low
**CR**																	
He2022(9)	Advanced Stage III or IV Melanoma	N+I	N/I	5	1605 (675/930)	OR	FE:2.12 [1.58;2.84] RE:3.21 [1.54;6.70]	FE:<0.0001 RE:0.0019	FE:70.2% RE:70.2%	0.2279	-1	-1	0	0	No	Low	Critically low
Menshawy 2018(2)	Advanced stage III or stage IV melanoma	N+I	I	2	771 (409/362)	RR	FE:5.75 [2.68;12.34] RE:5.75 [2.68;12.34]	FE:< 0.0001 RE:< 0.0001	FE:0.0% RE:0.0%	NA	-1	0	0	0	NA	Moderate	Critically low
**DCR**																	
Zeng2023(5)	Melanoma (include advanced melanoma and melanoma brain metastasis)	N+I	N/I/N+I Placebo	1	19(10/9)	RR	FE:1.08 [0.50;2.34] RE:1.08 [0.50;2.34]	FE:0.8450 RE:0.8450	FE:NA RE:NA	NA	-1	NA	0	-1	NA	Low	Critically low
**PRR**																	
Menshawy 2018(2)	Advanced stage III or stage IV melanoma	N+I	I	2	771 (409/362)	RR	FE:2.80 [2.16;3.63] RE:2.80 [2.16;3.63]	FE:<0.0001 RE:<0.0001	FE:0.0% RE:0.0%	NA	-1	0	0	0	NA	Moderate	Critically low

N:Nivolumab;I:Ipilimumab.OS:overall survival;ORR:objective response rate;PFS:progression-free survival;PRR:partial response rate;CR:complete response;DCR:disease control rate;OR*:overall response;HR:hazard ratio, OR:odds ratio;RR:relative risk;FE:fixed effect,RE:random effect;NA: not applicable; #: The research data could not be reanalyzed, so the original data were directly used.

Supplementary Table S2. Summary of the effectiveness outcomes of combined treatment with immune checkpoint inhibitors in patients, details of GRADE assessment and sensitivity analysis

Reference	Population	Intervention	Control	No. of studies	Sample size(Intervention /control)	Metric	Random Effect Size (95% CI)	*P-value*	I^2, %	Egger's Test (*P-value*)	Risk of Bias	Inconsistency	Indirectness	Imprecision	Publication	GRADE Rating	AMSTAR 2 Rating
**Mixed Adverse Reaction Outcomes**																	
Treatment Related Adverse Events																	
Hao2017#(8)	Advanced Cutaneous Melanoma	N+I	I	2	/	OR	FE:3.32<br>[2.44;4.51]<br>RE:3.32<br>[2.44;4.51]	FE:<0.0001<br>RE:<0.0001	FE:0.0%<br>RE: 0.0%	NA	-1	0	0	0	NA	Moderate	Critically low
He2022(9)	Advanced stage III or stage IV melanoma	N+I	N	4	/	OR	FE:3.46<br>[2.80;4.27]<br>RE:4.10<br>[1.67;10.04]	FE:<0.0001<br>RE:0.0020	FE:91.7%<br>RE:91.7%	0.8463	-1	-1	0	0	No	Low	Critically low
He2022(9)	Advanced stage III or stage IV melanoma	N+I	I	3	/	OR	FE:2.39<br>[1.85; 3.08]<br>RE:2.46<br>[0.86;7.04]	FE:<0.0001<br>RE: 0.0929	FE:93.0%<br>RE:93.0%	0.9825	-1	-1	0	0	No	Low	Critically low
Adverse Reactions																	
Li2019#(1)	Advanced or metastatic melanoma	N+I	N/I	/	1319	RR	1.13<br>[1.09, 1.17]<br>FE:/<br>RE:/	＜0.00001<br>FE:/<br>RE:/	FE:/<br>RE:/	NA	-1	NA	0	0	Yes	Low	Critically low
Zhang 2018(6)	Stage III or IV Melanoma	N+I	I	2	764<br>(407/357)	OR	FE:2.70<br>[1.51;4.82]<br>RE:1.74<br>[0.33;9.30]	FE:0.0008<br>RE:0.5169	FE:80.3%<br>RE:80.3%	NA	-1	-1	0	0	NA	Low	Critically low
Xu2021(3)	Advanced melanoma, Melanoma brain metastasis, Untreated melanoma	N+I	N	3	1320<br>(663/657)	RR	FE:1.88<br>[1.66;2.12]<br>RE:2.27<br>[1.42; 3.63]	FE:<0.0001<br>RE:0.0006	FE:87.7%<br>RE:87.7%	0.5248	-1	-1	0	0	No	Low	Critically low
Zeng2023(5)	Melanoma (include advanced melanoma and melanoma brain metastasis)	N+I	N/I/N+IPlacebo	6	3421<br>(1737/1684)	RR	FE:1.10<br>[1.08;1.13]<br>RE:1.10<br>[1.08;1.13]	FE:<0.0001<br>RE:<0.0001	FE:54.7%<br>RE:54.7%	0.9925	-1	-1	0	0	No	Low	Critically low

Zeng 2022#(7)	Melanoma, Melanoma brain metastasis	N+I	N	3	/	HR/ RR	FE:/ RE:1.16 [0.99; 1.36]	FE: / RE:0.07	FE:/ RE:71%	/	-1	-1	0	-1	NA	Critically low	Critically low
Treatment Discontinuation Rate																	
Zeng2023(5)	Melanoma (include advanced melanoma and melanoma brain metastasis)	N+I	N/I/N +IPlacebo	6	3417 (1737/1680)	RR	FE:3.07 [2.66; 3.54] RE:3.07 [2.66; 3.54]	FE:<0.0001 RE:<0.0001	FE:0.0% RE:0.0%	0.0688	-1	0	0	0	Yes	Low	Critically low
Zeng 2022#(7)	Melanoma, Melanoma brain metastasis	N+I	N	4	/	HR/ RR	FE:3.32 [2.79; 3.95] RE:/	FE: <0.01 RE:/	FE:0% RE:/	/	-1	0	0	0	NA	Moderate	Critically low
Mortality Rate																	
Zeng2023(5)	Melanoma (include advanced melanoma and melanoma brain metastasis)	N+I	N/I/N +IPlacebo	2	3085 (1542/1543)	RR	FE:2.93 [0.68;12.70] RE:2.93 [0.68;12.70]	FE:0.1513 RE:0.1513	FE:0.0% RE:0.0%	＜0.0001	-1	0	0	-1	No	Low	Critically low
Zeng 2022#(7)	Melanoma, Melanoma brain metastasis	N+I	N	4	/	HR/ RR	FE:4.34 [0.74; 25.54] RE:/	FE: 0.10 RE:/	FE:0% RE:/	/	-1	0	0	-1	NA	Low	Critically low
Adverse Events Leading to Treatment Cessation																	
Hao2017#(8)	Advanced Cutaneous Melanoma	N+I	I	2	/	OR	FE:2.90 [1.98;4.26] RE:2.90 [1.98;4.26]	FE:<0.0001 RE:<0.0001	FE:0.0% RE:0.0%;	NA	-1	0	0	0	NA	Moderate	Critically low
**Gastrointestinal Adverse Reaction Outcomes**																	
Gastrointestinal Adverse Reactions																	
Pradeep 2022(10)	Advanced stage III or stage IV melanoma	N+I	I	6	2783 (1443/1340)	RR	FE:1.29 [1.20;1.39] RE:1.29 [1.20;1.39]	FE:<0.0001 RE:<0.0001	FE:0.0% RE:0.0%	0.0611	-1	0	0	0	Yes	Low	Critically low
Pradeep 2022(10)	Advanced stage III or stage IV melanoma	N+I	N	7	2610 (1309/1301)	RR	FE:2.36 [2.12; 2.62] RE:2.35 [2.08; 2.64]	FE:<0.0001 RE:<0.0001	FE:52.1% RE:52.1%	0.6249	-1	-1	0	0	No	Low	Critically low
Nausea																	
Li2019#(1)	Advanced or metastatic melanoma	N+I	N/I	/	1401	RR	1.83 [1.47, 2.26] FE:/ RE:/	＜0.00001 FE:/ RE:/	FE:/ RE:/	/	-1	NA	0	0	Yes	Low	Critically low

Zeng2023(5)	Melanoma melanoma	N+I	N/I/N +IPlacebo	5	3398 (1727/1671)	RR	FE:1.56 [1.34;1.83] RE:1.59 [1.27;1.99]	FE:<0.0001 RE:<0.0001	FE:25.7% RE:25.7%	0.7639	-1	0	0	0	No	Moderate	Critically low
Hao2017#(8)	Advanced Cutaneous Melanoma	N+I	I	/	/	OR	2.30 [0.62;8.56] FE:/ RE:/	FE:/ RE:/	FE:/ RE:/	/	-1	NA	0	-1	Yes	Critically low	Critically low
Diarrhea																	
Li2019#(1)	Advanced or metastatic melanoma	N+I	N/I	/	1416	RR	1.53 [0.96, 2.43] FE:/ RE:/	0.07 FE:/ RE:/	FE:/ RE:/	/	-1	NA	0	-1	Yes	Critically low	Critically low
Zeng2023(5)	Melanoma (include advanced melanoma and melanoma brain metastasis)	N+I	N/I/N +IPlacebo	5	3359 (1704/1655)	RR	FE:1.45 [1.30; 1.62] RE:1.48 [1.22; 1.79]	FE:<0.0001 RE:<0.0001	FE:45.8% RE:45.8%	0.7663	-1	0	0	0	No	Moderate	Critically low
Hao2017#(8)	Advanced Cutaneous Melanoma	N+I	I	/	/	OR	0.32 [0.21;0.49] FE:/ RE:/	FE:/ RE:/	FE:/ RE:/	/	-1	NA	0	0	Yes	Low	Critically low
Colitis																	
Li2019#(1)	Advanced or metastatic melanoma	N+I	N/I	/	1401	RR	2.54 [0.81, 8.01] FE:/ RE:/	0.11 FE:/ RE:/	FE:/ RE:/	/	-1	NA	0	-1	Yes	Critically low	Critically low
Zeng2023(5)	Melanoma (include advanced melanoma and melanoma brain metastasis)	N+I	N/I/N +IPlacebo	6	3302 (1679/1623)	RR	FE:1.90 [1.44; 2.51] RE:2.08 [1.08; 4.01]	FE:<0.0001 RE:0.0292	FE:77.5% RE:77.5%	0.8558	-1	-1	0	0	No	Low	Critically low
Hao2017#(8)	Advanced Cutaneous Melanoma	N+I	I	/	/	OR	1.12 [0.67;1.86] FE:/ RE:/	FE:/ RE:/	FE:/ RE:/	/	-1	NA	0	-1	High	Critically low	Critically low
Vomiting																	
Li2019#(1)	Advanced or metastatic melanoma	N+I	N/I	/	1463	RR	1.69 [1.23, 2.33] FE:/ RE:/	0.001 FE:/ RE:/	FE:/ RE:/	/	-1	NA	0	0	Yes	Low	Critically low

Zeng2023(5)	Melanoma (include advanced melanoma and melanoma brain metastasis)	N+I	N/I/N +IPlacebo	2	1397 (720/677)	RR	FE:2.07 [1.51; 2.84] RE:2.07 [1.51; 2.84]	FE:<0.0001 RE:<0.0001	FE:0.0% RE:0.0%	0.7852	0	0	0	0	No	High	Critically low
Hao2017# (8)	Advanced Cutaneous Melanoma	N+I	I	/	/	OR	5.46 [0.99;30.06] FE:/ RE:/	FE:/ RE:/	FE:/ RE:/		-1	NA	0	-1	Yes	Critically low	Critically low
Decreased Appetite																	
Zeng2023(5)	Melanoma (include advanced melanoma and melanoma brain metastasis)	N+I	N/I/N +IPlacebo	2	1397 (720/677)	RR	FE:1.57 [1.22; 2.04] RE:1.57 [1.22; 2.04]	FE:0.0006 RE:0.0006	FE:0.0% RE:0.0%	0.7562	0	0	0	0	No	High	Critically low
Hao2017# (8)	Advanced Cutaneous Melanoma	N+I	I	/	/	OR	4.01 [0.45;36.11] FE:/ RE:/	FE:/ RE:/	FE:/ RE:/	/	-1	NA	0	-1	Yes	Critically low	Critically low
Abdominal Pain																	
Hao2017# (8)	Advanced Cutaneous Melanoma	N+I	I	/	/	OR	0.16 [0.01;4.02] FE:/ RE:/	FE:/ RE:/	FE:/ RE:/	/	-1	NA	0	-1	Yes	Critically low	Critically low
Constipation																	
Hao2017# (8)	Advanced Cutaneous Melanoma	N+I	I	/	/	OR	1.49 [0.06;37.34] FE:/ RE:/	FE:/ RE:/	FE:/ RE:/	/	-1	NA	0	-1	Yes	Critically low	Critically low
**Liver Adverse Reaction Outcomes**																	
Liver Adverse Reactions																	
Pradeep 2022(10)	Advanced stage III or stage IV melanoma	N+I	I	6	2782 (1442/1340)	RR	FE:4.58 [3.75;5.58] RE:4.58 [3.75;5.58]	FE:<0.0001 RE:<0.0001	FE:0.0% RE:0.0%	0.3435	-1	0	0	0	No	Moderate	Critically low
Pradeep 2022(10)	Advanced stage III or stage IV melanoma	N+I	N	6	1984 (996/988)	RR	FE:4.04 [3.24; 5.04] RE:4.04 [3.24;5.04]	FE:<0.0001 RE:<0.0001	FE:0.0% RE:0.0%	0.2889	-1	0	0	0	No	Moderate	Critically low
Elevated Liver Function Test Values																	

Li2019#(1)	Advanced or metastatic melanoma	N+I	N/I	/	1319	RR	4.31 [3.25, 5.71] FE:/ RE:/	＜0.00001 FE:/ RE:/	FE:/ RE:/	/	-1	NA	0	0	Yes	Critically low	Critically low
Incidence of AST Elevation																	
Zeng2023(5)	Melanoma (include advanced melanoma and melanoma brain metastasis)	N+I	N/I/N+IPlacebo	5	3359 (1704/1655)	RR	FE:3.77 [2.77; 5.12] RE:3.77 [2.77; 5.12]	FE:<0.0001 RE:<0.0001	FE: 0.0% RE:0.0%	0.4648	-1	0	0	0	No	Moderate	Critically low
Hao2017#(8)	Advanced Cutaneous Melanoma	N+I	I	/	/	OR	9.49 [2.56;35.19] FE:/ RE:/	FE:/ RE:/	FE:/ RE:/		-1	NA	0	-1	Yes	Critically low	Critically low
Incidence of ALT Elevation																	
Zeng2023(5)	Melanoma (include advanced melanoma and melanoma brain metastasis)	N+I	N/I/N+IPlacebo	5	3357 (1702/1655)	RR	FE:4.07 [3.02;5.48] RE:4.07 [3.02;5.48]	FE:<0.0001 RE:<0.0001	FE:0.0% RE:0.0%	0.0442	-1	0	0	0	Yes	Low	Critically low
Hao2017#(8)	Advanced Cutaneous Melanoma	N+I	I	/	/	OR	5.54 [2.10;14.63] FE:/ RE:/	FE:/ RE:/	FE:/ RE:/	/	-1	NA	0	-1	Yes	Critically low	Critically low
**Skin Adverse Reaction Outcomes**																	
Rash																	
Li2019#(1)	Advanced or metastatic melanoma	N+I	N/I	/	202	RR	2.07 [0.87, 4.93] FE:/ RE:/	0.1 FE:/ RE:/	FE:/ RE:/	/	-1	NA	0	-1	Yes	Critically low	Critically low
Zeng2023(5)	Melanoma (include advanced melanoma and melanoma brain	N+I	N/I/N+IPlacebo	5	3401(1730/1671)	RR	FE:1.25 [1.08;1.45] RE:1.52 [1.07;2.15]	FE:0.0035 RE:0.0194	FE:75.5% RE:75.5%	0.0537	-1	-1	0	0	Yes	Critically low	Critically low
Hao2017#(8)	Advanced Cutaneous Melanoma	N+I	I	/	/	OR	2.87 [1.16;7.11] FE:/ RE:/	FE:/ RE:/	FE:/ RE:/	/	-1	NA	0	-1	Yes	Critically low	Critically low
Itching																	

Li2019#(1)	Advanced or metastatic melanoma	N+I	N/I	/	202	RR	2.06 [0.57, 7.45] FE:/ RE:/	0.27 FE:/ RE:/	FE:/ RE:/	/	-1	NA	0	-1	Yes	Critically low	Critically low
Zeng2023(5)	Melanoma (include advanced melanoma and melanoma brain	N+I	N/I/N +IPlacebo	6	3417 (1737/1680)	RR	FE:1.43 [1.25;1.65] RE:2.38 [0.95;5.98]	FE:<0.0001 RE:0.0647	FE:94.8% RE:94.8%	0.2648	-1	-1	0	0	No	Low	Critically low
Hao2017#(8)	Advanced Cutaneous Melanoma	N+I	I	/	/	OR	4.23 [0.74;24.12] FE:/ RE:/	FE:/ RE:/	FE:/ RE:/	/	-1	NA	0	-1	Yes	Critically low	Critically low
Skin Adverse Reactions																	
Pradeep 2022(10)	Advanced stage III or stage IV melanoma	N+I	I	6	2154 (1129/1025)	RR	FE:1.12[1.06;1.19] RE:1.12[1.06;1.19]	FE:<0.0001 RE:<0.0001	FE:0.0% RE:0.0%	0.0157	-1	0	0	-1	No	Moderate	Critically low
Pradeep 2022(10)	Advanced stage III or stage IV melanoma	N+I	N	7	1961 (985/976)	RR	FE:1.50 [1.40;1.61] RE:1.51 [1.40;1.62]	FE:<0.0001 RE:<0.0001	FE:36.3% RE:36.3%	0.2590	-1	0	0	0	No	Moderate	Critically low
Maculopapular Rash																	
Hao2017#(8)	Advanced Cutaneous Melanoma	N+I	I	/	/	OR	3.56 [0.18;70.33] FE:/ RE:/	FE:/ RE:/	FE:/ RE:/	/	-1	NA	0	-1	Yes	Critically low	Critically low
**Pulmonary Adverse Reaction Outcomes**																	
Pulmonary Adverse Reactions																	
Pradeep 2022(10)	Advanced stage III or stage IV melanoma	N+I	I	5	2154 (1129/1025)	RR	FE:4.21 [2.55;6.94] RE:4.21 [2.55;6.94]	FE:<0.0001 RE:<0.0001	FE:0.0% RE:0.0%	0.5174	-1	0	0	0	No	Moderate	Critically low
Pradeep 2022(10)	Advanced stage III or stage IV melanoma	N+I	N	5	1961 (985/976)	RR	FE:4.22 [2.50; 7.12] RE:4.22 [2.50; 7.12]	FE:<0.0001 RE:<0.0001	FE:0.0% RE:0.0%	0.0559	-1	0	0	0	Yes	Low	Critically low
Pneumonia																	
Zeng2023(5)	Melanoma (include advanced melanoma and melanoma brain metastasis)	N+I	N/I/N +IPlacebo	5	3398 (1727/1671)	RR	FE:3.00 [1.96; 4.60] RE:3.02 [1.96; 4.67]	FE:<0.0001 RE:<0.0001	FE:0.7% RE:0.7%	0.2578	-1	0	0	0	No	Moderate	Critically low

Hao2017# (8)		N+I	I	/	/	OR	0.98 [0.09;11.08] FE:/ RE:/	FE:/ RE:/	FE:/ RE:/		-1	NA	0	-1	Yes	Critic ally low	Critically low
Dyspnea																	
Hao2017# (8)	Advanced Cutaneous Melanoma	N+I	I	/	/	OR	4.19 [0.49;35.41] FE:/ RE:/	FE:/ RE:/	FE:/ RE:/	/	-1	NA	0	-1	Yes	Critic ally low	Critically low
**Endocrine Adverse Reaction Outcomes**																	
Endocrine Adverse Reactions																	
Pradeep 2022(10)	Advanced stage III or stage IV melanoma	N+I	I	6	2782 (1442/1340)	RR	FE:3.19[2.6 6; 3.83] RE:3.07[2.2 6; 4.19]	FE:<0.0001 RE:<0.0001	FE:55.0% RE:55.0%	0.5910	-1	-1	0	0	No	Low	Critically low
Pradeep 2022(10)	Advanced stage III or stage IV melanoma	N+I	N	7	2600 (1309/1291)	RR	FE:2.24[1.9 2;2.63] RE:2.24[1.9 2;2.63]	FE:<0.0001 RE:<0.0001	FE:0.0% RE:0.0%	0.7713	-1	0	0	0	No	Mod erate	Critically low
Hypothyroidism																	
Li2019#(1)	Advanced or metastatic melanoma	N+I	N/I	/	1319	RR	2.66 [1.24, 5.69] FE:/ RE:/	0.01 FE:/ RE:/	FE:/ RE:/	/	-1	NA	0	0	Yes	Low	Critically low
Zeng2023(5)	Melanoma (include advanced melanoma and melanoma brain metastasis)	N+I	N/I/N +IPlac ebo	4	3338 (1692/1646)	RR	FE:1.71 [1.46;2.01] RE:1.89 [1.26;2.83]	FE:<0.0001 RE:0.0022	FE:69.5% RE:69.5%	0.6168	0	-1	0	0	No	Mod erate	Critically low
Hao2017# (8)	Advanced Cutaneous Melanoma	N+I	I	/	/	OR	2.99 [0.12 ;73.96] FE:/ RE:/	FE:/ RE:/	FE:/ RE:/	/	-1	NA	0	-1	Yes	Critic ally low	Critically low
Hypopituitarism																	
Li2019#(1)	Advanced or metastatic melanoma	N+I	N/I	/	1259	RR	5.02 [1.06,23.82] FE:/ RE:/	0.04 FE:/ RE:/	FE:/ RE:/	/	-1	NA	0	-1	Yes	Critic ally low	Critically low
Pituitary Inflammation																	

Li2019#(1)	Advanced or metastatic melanoma	N+I	N/I	/	1259	RR	4.24 [0.68,26.33] FE:/ RE:/	0.12 FE:/ RE:/	FE:/ RE:/	/	-1	NA	0	-1	Yes	Critically low	Critically low
Hao2017#(8)	Advanced Cutaneous Melanoma	N+I	I	/	/	OR	0.48 [0.07, 3.51] FE:/ RE:/	FE:/ RE:/	FE:/ RE:/	/	-1	NA	0	-1	Yes	Critically low	Critically low
Hyperthyroidism																	
Zeng2023(5)	Melanoma (include advanced melanoma and melanoma brain metastasis)	N+I	N/I/N+IPlacebo	3	3143 (1577/1566)	RR	FE:1.92 [1.55;2.36] RE:3.10 [1.28;7.51]	FE:<0.0001 RE:0.0122	FE:71.8% RE:71.8%	0.2040	-1	-1	0	0	No	Low	Critically low
**Hematological Adverse Reaction Outcomes**																	
Hematological Adverse Reactions																	
Pradeep 2022(10)	Advanced stage III or stage IV melanoma	N+I	I	4	2012 (1034/978)	RR	FE:0.98 [0.78;1.24] RE:0.98 [0.78;1.24]	FE:0.8689 RE:0.8689	FE:0.0% RE:0.0%	< 0.0001	-1	0	0	-1	Yes	Critically low	Critically low
Pradeep 2022(10)	Advanced stage III or stage IV melanoma	N+I	N	5	1920 (960/960)	RR	FE:2.33 [1.70;3.19] RE:2.33 [1.70;3.19]	FE:<0.0001 RE:<0.0001	FE:0.0% RE:0.0%	0.5846	-1	0	0	0	No	Moderate	Critically low
**Others**																	
Fatigue																	
Li2019#(1)	Advanced or metastatic melanoma	N+I	N/I	/	1461	RR	1.13 [0.88, 1.46] FE:/ RE:/	0.34 FE:/ RE:/	FE:/ RE:/	/	-1	NA	0	-1	Yes	Critically low	Critically low
Zeng2023(5)	Melanoma (include advanced melanoma and melanoma brain metastasis)	N+I	N/I/N+IPlacebo	5	3398 (1727/1671)	RR	FE:1.08 [0.98; 1.19] RE:1.12 [0.94; 1.34]	FE:0.1040 RE:0.1988	FE:55.9% RE:55.9%	0.4046	-1	-1	0	-1	No	Critically low	Critically low
Hao2017#(8)	Advanced Cutaneous Melanoma	N+I	I	/	/	OR	4.68 [1.46;14.96] FE:/ RE:/	FE:/ RE:/	FE:/ RE:/	/	-1	NA	0	-1	Yes	Critically low	Critically low
Fever																	

Hao2017# (8)	Advanced Cutaneous Melanoma	N+I	I	/	/	OR	2.61 [0.41 ;16.65] FE:/ RE:/	FE:/ RE:/	FE:/ RE:/	/	-1	NA	0	-1	Yes	Critically low	Critically low
Headache																	
Hao2017# (8)	Advanced Cutaneous Melanoma	N+I	I	/	/	OR	1.59 [0.22;11.55] FE:/ RE:/	FE:/ RE:/	FE:/ RE:/	/	-1	NA	0	-1	Yes	Critically low	Critically low
Elevated Lipase																	
Hao2017# (8)	Advanced Cutaneous Melanoma	N+I	I	/	/	OR	4.19 [0.51;34.52] FE:/ RE:/	FE:/ RE:/	FE:/ RE:/	/	-1	NA	0	-1	Yes	Critically low	Critically low
Arthralgia																	
Hao2017# (8)	Advanced Cutaneous Melanoma	N+I	I	/	/	OR	2.99 [0.12;73.69] FE:/ RE:/	FE:/ RE:/	FE:/ RE:/	/	-1	NA	0	-1	Yes	Critically low	Critically low

N:Nivolumab;I:Ipilimumab;HR:hazard ratio;OR:odds ratio;RR:relative risk;FE:fixed effect;RE: random effect;NA: not applicable;#: The research data could not be reanalyzed, so the original data were directly used.

Supplementary Table S3. Summary of adverse reactions in patients treated with immune checkpoint inhibitors in combination, details of GRADE assessment and sensitivity analysis

References

1.Li J, Gu J. Efficacy and safety of ipilimumab for treating advanced melanoma: A systematic review and meta-analysis. J Clin Pharm Ther. 2019;44(3):420–9.
2.Menshawy A, Eltonob AA, Barkat SA, Ghanem A, Mniesy MM, Mohamed I, et al. Nivolumab monotherapy or in combination with ipilimumab for metastatic melanoma: systematic review and meta-analysis of randomized-controlled trials. Melanoma Res. 2018;28(5):371–9.
3.Xu Y, Hezam K, Ali MG, Wang Y, Zhang J. The efficacy and safety of Nivolumab combined with Ipilimumab in the immunotherapy of cancer: a meta-analysis. Immunopharmacol Immunotoxicol. 2021;43(3):386–94.
4.Weng, Y.; Chen, L.; Song, Q.; Peng, M. The efficacy and safety of solid tumors in combination therapy with immune checkpoint 458 inhibitor: A systematic review. *Mod. Oncol.* 2020, *28*, 121–130. https://doi.org/10.3969/j.issn.1672-4992.2020.01.030.
5. Zeng, J. Meta-Analysis of the Efficacy and Safety of anti-PD-1/PD-L1 Combined with CTLA-4 Inhibitors in the Treatment of Solid. Master's Thesis, Dali University, Dali City, China, 2023.
6. Zhang, C. Efficacy and Safety of Anti PD-1 Antibodies in the Treatment of Advanced Melanoma: A Meta-Analysis. Master's Thesis, University of South China, Hengyang,China, 2018.
7. Zeng, C.R.; Li, H.; Wen, J. Meta-analysis on the efficacy and safety of PD-1/PD-L1 and CTLA-4 combination therapy in the treatment of solid tumor patients. *Chin. J. Hosp. Pharm.* 2022, *42*, 1808–1816 https://doi.org/10.13286/j.1001-5213.2022.17.13.
8.Hao C, Tian J, Liu H, Li F, Niu H, Zhu B. Efficacy and safety of anti-PD-1 and anti-PD-1 combined with anti-CTLA-4 immunotherapy to advanced melanoma: A systematic review and meta-analysis of randomized controlled trials. Medicine (Baltimore). 2017;96(26):e7325.

9.He R, Zhao X, Liu J, Zhou Y, Zhang X, Cheng F. PD-1 and CTLA-4 inhibitors in combination vs. alone for the treatment of advanced melanoma: A systematic review and meta-analysis. Medicine (Baltimore). 2022;101(41):e30561.
10.Pradeep J, Win TT, Aye SN, Sreeramareddy CT. Efficacy and Safety of Immune Checkpoint Inhibitors for Advanced Malignant Melanoma: A Meta-Analysis on Monotherapy Vs Combination Therapy. J Cancer. 2022;13(10):3091–102.
